# High-Level CNN and Machine Learning Methods for Speaker Recognition

**DOI:** 10.3390/s23073461

**Published:** 2023-03-25

**Authors:** Giovanni Costantini, Valerio Cesarini, Emanuele Brenna

**Affiliations:** Department of Electronic Engineering, University of Rome Tor Vergata, 00133 Roma, Italy

**Keywords:** speaker recognition, CNN, AlexNet, Naïve Bayes, Machine Learning, audio, F0

## Abstract

Speaker Recognition (SR) is a common task in AI-based sound analysis, involving structurally different methodologies such as Deep Learning or “traditional” Machine Learning (ML). In this paper, we compared and explored the two methodologies on the DEMoS dataset consisting of 8869 audio files of 58 speakers in different emotional states. A custom CNN is compared to several pre-trained nets using image inputs of spectrograms and Cepstral-temporal (MFCC) graphs. AML approach based on acoustic feature extraction, selection and multi-class classification by means of a Naïve Bayes model is also considered. Results show how a custom, less deep CNN trained on grayscale spectrogram images obtain the most accurate results, 90.15% on grayscale spectrograms and 83.17% on colored MFCC. AlexNet provides comparable results, reaching 89.28% on spectrograms and 83.43% on MFCC.The Naïve Bayes classifier provides a 87.09% accuracy and a 0.985 average AUC while being faster to train and more interpretable. Feature selection shows how F0, MFCC and voicing-related features are the most characterizing for this SR task. The high amount of training samples and the emotional content of the DEMoS dataset better reflect a real case scenario for speaker recognition, and account for the generalization power of the models.

## 1. Introduction

The automatic analysis of vocal signals is an ever-growing topic within Artificial Intelligence (AI), since the voice contains a very deep array of information about the subjects and their state and it is the most common real-world vector used in telecommunications, all the while leading to completely non-invasive assessments [1]. AI-enhanced voice analysis employs expert models based either on high-level implementations such as Deep Learning (DL) or more case-specific pipelines such as those seen in “traditional” Machine Learning (ML), with the common aim to identify characteristic features for the identification of peculiarities within the voice, for tasks such as pathology detection [2,3,4], emotion recognition [5,6], etc. 

Speaker Recognition (SR) is widely used application of voice analysis that can be foundational for many modern applications, such as vocal controls in smartphones or virtual assistant technologies (for domotics or car control). A specific case of SR sometimes referred to as Speaker Identification is aimed at recognizing who is talking among a pool of known people, meanwhile Speaker Verification is centered on confirming the identity of a predefined speaker and, being an inherently binary yes/no task, does not need to rely on a known pool of people. Both techniques are either faced in a text-dependent way, with datasets of speakers uttering predefined sentences, or text-independent, which, albeit less pinpoint-accurate, is often the only possible solution in many practical applications. Hybrid solutions are employed by segmenting text-dependent datasets into fragmented, a-semantic and text-independent sets (audio file belongs to a specific person). Non-semantic SR saw promising results when ML models were trained on acoustic features representative of phonetic and prosodic characteristics that in turn generate a “voice model” for each speaker [7]. The “usual” pipeline for SR involves feature extraction and selection, feature modeling and, at last, the building of a “speaker model”. In the previous decade, Gaussian Mixture Models (GMM) [8] and several classifiers such as Support Vector Machines (SVM) [9] often provided state-of-the-art results, deriving the concept of i-vectors as low-level representatives of inter and intra-speaker characteristics that build the speaker model [10]. Commonly employed acoustic features usually involve Mel-Frequency Cepstral Coefficients (MFCC) [11], more than pitch-related measures such as Fundamental Frequency (F0). With the ever-growing usage of DL, there is the introduction of the concept of x-vectors, which are basically deep features that constitute the speaker model within DL algorithms for SR [12]. 

Amongst the relevant works, [13] used MFCC and Sub-band Cepstral coefficients to obtain a 95% accuracy on a small sample of 20 speakers; [14] employed a Convolutional Neural Network (CNN) on a dataset of unspecified cardinality without overcoming 80% accuracy; [15] adapted probabilistic decision with Bayesian Learning on DL; while [16] developed a CNN-based for identifying tonal speech sentences and adding instrumental knowledge, leading to an 89.15% accuracy and a 10.56% WER for continuous and extensive vocabulary sentences of speech signals.

With these premises, the present work is based on the comparison of high-level AI methodologies for SR that do not involve visible speaker modeling, and that encompass the two main realms of algorithms, being DL and ML. A well recorded dataset of many utterances that also encompass several different emotions is faced in a text-independent way and multi-class classification tasks are prepared for SR. Several DL architectures are experimented with different feature inputs, and the results are also compared to a ML pipeline trained on an algorithmically selected subset of acoustic features that encompass the vast majority of the common domains in voice analysis. The features assessed as most relevant are reported, showing that there are many alternatives and possible improvements over MFCC. 

## 2. Materials and Methods

The following section will describe the dataset used (DEMoS: Database of Elicited Mood in Speech) as well as the AI approaches used for SR. The main focus is exploring and comparing DL approaches, involving tests on several CNN architectures and reporting results of a custom one and a pre-trained one, versus a traditional ML pipeline based on acoustic features.

### 2.1. Dataset

The present study employs the DEMoS dataset [17], which is a corpus of induced emotional speech in Italian consisting of audio data gathered by an initial population of 68 people reading several pre-defined texts with the elicitation of 7 different emotional states plus neutral. The 68 participants (23 females, 45 males) were all University students (mean age 23.7 years, std.dev. 4.3 years), and the initial corpus encompasses 9697 audio samples: 3444 produced by females (3332 with an emotional content and 112 neutral); 6253 produced by males (6033 with an emotional content and 220 neutral). A total of 8 states, namely guilt, disgust, joy, fear, rage, surprise, sadness and neutral were professionally induced using Mood Induction Procedures (MIP) accompanied by an alexithymia test (which assesses the ability of a person to recognize their and others’ emotions), a self-assessment (to check if the speaker recognizes the emotion that has been elicited) and a posterior annotation with experts checking the speaker emotion to be the same as the one elicited. Specifically, the emotions happiness, surprise, guilt and sadness were elicited with the combination of MIP, involving listening to music, autobiographic recall, reading sentences associated to a given emotional state and reading a full text aimed at inducing a certain emotion. The emotions anger and fear were induced with the full text MIP, the single sentence MIP and the vision of a short film; the emotion disgust was elicited through the single sentence MIP, the full text MIP and the visualization of images associated to disgust. More information on the elicitation methods and their content as well as inclusion/exclusion criteria is given in the DEMoS paper [17]. Audio was recorded from the full text readings and single sentences; every task was manually segmented according to syntax and prosody, removing long silences, resulting in small, text-independent segments of a mean duration of 2.9 s. 

**Figure 1:**
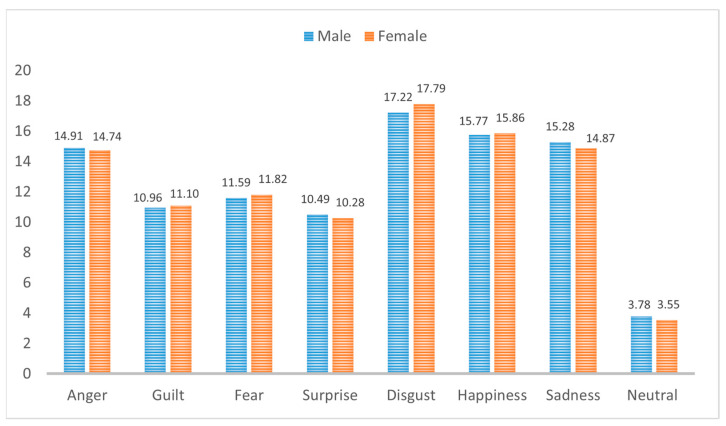
Percentual distribution of each emotion with respect to the global number of instances for male (blue) and female (orange) speakers.

From the original 68 people, 58 speakers (19 female, 29 male) were retained, for a total of 8869 segments (3046 by females, 5823 by males), after applying exclusion criteria based on self-perception, external annotation of the detectable presence of a given emotion, and fluency in speech. The number of instances (segments) for each speaker, divided by sex, and the distribution of each emotion is shown in Table 1, while Figure 1 displays the percentual distribution of each emotion versus the total for each sex. This shows how the emotional speech is balanced between male and female speakers, and almost balanced among emotions, with neutral samples being underrepresented.

**Figure 2:**
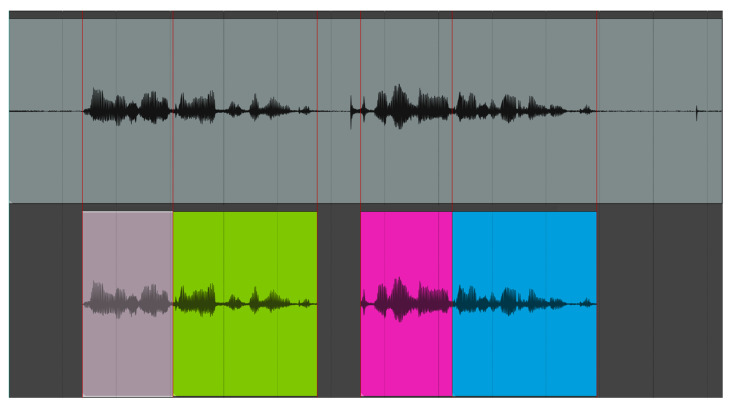
Example of segmentation starting from the original signal (above, in grey). The segments are defined manually, according to prosody and syntax, and may contain full words or parts. Silence at the beginning and end, long pauses and eventual noises are all removed. The individual segments, divided on the time points defined by the red lines, are found below (each is differently colored).

**Figure 3:**
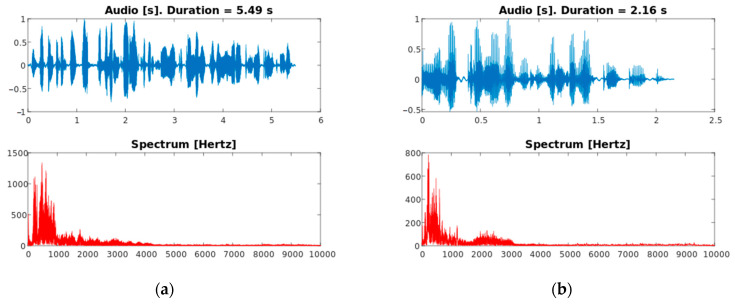
(**a**) Example audio segment from a female speaker, with emotion “neutral”; (**b**) Example audio segment from a male speaker, with emotion “guilt”.

The DEMoS dataset was chosen for SR due to its variability in terms of tone and context given by the emotions, and it was prepared for text-independent SR thanks to the prosodic and syntactic segmentation. All files have been recorded with the aid of a professional microphone in an indoor environment within a small room without background noises, additional voices or machinery, are formatted as PCM .wav with a sampling frequency of 48 kHz and 16 bit of depth and have been normalized to a unitary peak amplitude. In each audio sample resulted from the segmentation procedure, one or more full words can be found as well as parts of a word, divided according to syllables. An example of (a part of) the original signal and the segments derived from it is reported in Figure 2, and an example of two audio segments as found in the final dataset, by a male and a female speaker, is provided in Figure 3 along with spectrums up to 10 kHz. 

### 2.2. CNN-Based Deep Learning Approach

A Convolutional Neural Network (CNN) is an artificial neural network that is specifically designed to process data with a grid-like topology; it is a deep learning architecture that has been highly successful in various image and video processing tasks, such as object recognition or image classification. CNN are mostly employed on images in reason of their filtering nature; they effectively identify local graphical features, which in turn makes them well-suited to treat spectrograms, especially in speech tasks since fundamental frequency, harmonics and formants appear as graphically localized lines. Therefore, even for audio applications, graphic plots (images) are preferred as inputs.

Convolutional Neural Networks (CNN) and DL have gradually become standards for voice analysis; however, as suggested by Cummins et al. in [18], for some tasks DL could still be surpassed by traditional ML, especially due to limitations such as the complexity of acoustic features or the scarcity of datasets.

We experimented custom architectures as well as pre-trained ones, with two different image inputs: the commonly employed Mel-spectrograms, and the less explored Mel-Frequency Cepstral Coefficients (MFCC) as a matrix (Supplementary Materials).

All of the CNN-based approaches have been implemented on MATLAB^®^ (by Mathworks, Inc., Natick, MA, USA).

#### 2.2.1. Feature Maps: Fourier Spectrograms and MFCC

The most commonly employed image input for CNNs in audio recognition tasks is the spectrogram, which is a visual representation of the frequency spectrum of a signal, computed by means of a sliding window STFT, as it varies with time. It is rendered as a 2D plot with the x-axis representing time, the y-axis representing frequency, and the color representing amplitude. 

Since relevant information in vocal signals can usually be found below 10 kHz [19], in order to reduce the computational complexity and to better localize relevant features in the visualization of the spectrogram, all of the audio signals were re-sampled at half the original frequency (24 kHz). A window of 2048 samples with an overlap ratio of 50% was used for the Fourier transform, implemented by a 1024-point FFT. Colored spectrograms and their grayscale counterpart were then generated and saved as .png images. Spectrograms contain both frequency and time-related information, which allow the net to train on the spectrum as well as the temporal evolution of the audio segment. 

Additionally, an alternative representation was also used: Mel-Frequency Cepstral Coefficients (MFCC). They are based on the concept of Cepstrum [20], which is the (inverse) Fourier transform of the spectrum. MFCC specifically are derived from applying a perceptual scaling called Mel [21], which re-weighs frequencies according to a discrete filter-bank based on the auditory principle of “tones” being equally spaced in frequency, according to the formula:

(1) $$m_{n}=2595\cdot \log_{10}\left(1+\frac{f_{n}}{700}\right)$$

where $m_{n}$ is the n-th MFCC associated to the n-th frequency band $f_{n}$. The process of calculating MFCCs consist of Fourier-transforming a signal, using triangular-shaped filters to isolate each Mel frequency component, then applying the logarithm obtaining a Log-scale Power Spectral Density and finally applying the Discrete Cosine Transform. Because the MFCCs are based on the logarithm of the filter-bank output, they are relatively insensitive to the overall level of the signal and are more representative of the spectral envelope of the signal and of pitch.

**Figure 4:**
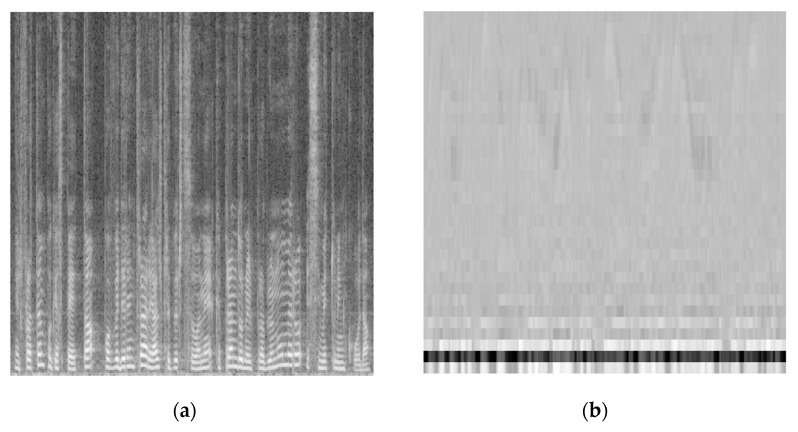
(**a**) Grayscale spectrogram; (**b**) Grayscale MFCC of the same audio segment.

The number of discrete Mel bands chosen (number of MFCC) was 32. Temporal windowing was applied to derive temporal-cepstral graphs, with each MFCC representing one y-axis bin. For such graphs, colored and grayscale images were produced. An example of grayscale spectrograms and MFCC-graphs as they were used to train the nets is given in Figure 4. 

#### 2.2.2. Architectures

**Figure 5:**
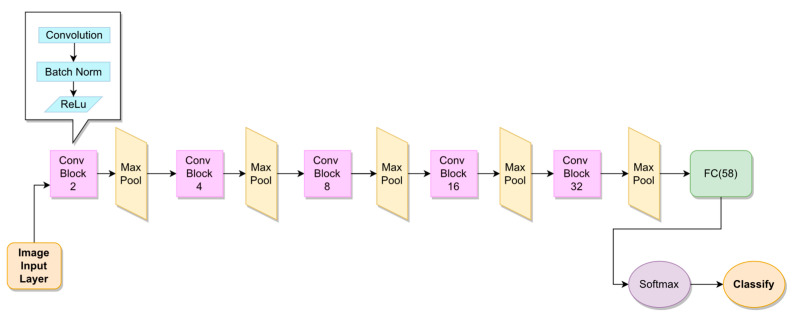
CNN1 architecture. Each “Conv Block”, in pink, is comprised of a convolutional block, a batch normalization layer and a ReLu activation function. The number in each block corresponds to the number of filters/neurons in the corresponding each layer: as an example, the first “Conv Block” (pink) embeds a convolutional layer of 2 filters, the second embeds a convolutional layer of 4 filters, etc. The block labeled “FC(58)” (green) refers to a fully-connected layer with 58 neurons.

Several CNN architectures were experimented using either spectrograms or MFCC-graphs as image inputs. Besides custom ones, common pre-trained nets were also employed with the aid of transfer learning, namely GoogleNet [22] and AlexNet [23] pre-trained on the ImageNet dataset. The results only report those that brought significant results, which are the AlexNet and our custom architecture, which will be referred to as “CNN1” and that we built with the aim to be less deep without sacrificing accuracy. CNN1 consists of five convolutional layers of increasing number of filters, from 2 to 32. Each convolutional layer is followed by a batch normalization layer, a ReLu activation function and a max-pooling layer. A fully connected layer of 58 neurons, one per speaker, flattens the convolutional output and brings to the classification which is rendered by a softmax layer followed by a simple decision layer. The CNN1 architecture is detailed in Figure 5. 

The net was trained for a fixed number of epochs equal to 100, with an initial learning rate of 0.001 and a stochastic gradient descent (SGDM) optimizer with a momentum equal to 0.9. The AlexNet, composed of 8 convolutional layers, was optimized with a SGDM algorithm; a learning rate of 0.0001 was used for the 6 outer layers, more prone to identifying “general” graphic characteristics, while a learning rate of 0.01 was used for the last 2 layers, which adapt more to the new training data. This piecewise learning rate in transfer-learnt nets, especially when deep, allows outer layers to only slightly adapt to the new input, since they are already pre-trained on similar data and are inherently producing macroscopic features anyway; on the other hand, the inner layers produce very specific features that allow for classification, and need to learn the new input much more closely, hence the higher learning rate. All CNNs were trained using a 80–20 holdout procedure for each speaker balanced for each emotion. Since emotional content can alter key vocal/spectral characteristics, in turn biasing the net, the distribution of the training and validation set in terms of emotional speech was the same by using 20% of each speaker’s samples for each emotion to form the validation set. This procedure still resulted in an 80–20 division, with the training set having 7095 samples and the validation set having 1774 samples. Trained nets were run on the never before-seen validation set and multi-class accuracy was subsequently computed. 

### 2.3. Machine Learning-Based Approach

For the Machine Learning (ML) approach, a pipeline composed of the following steps was built:

1. Feature Extraction;
2. Feature Selection;
3. Classifier training.

#### 2.3.1. Feature Extraction and Selection 

From each audio segment, a feature matrix was derived by means of feature extraction, with the tool OpenSMILE^®^ (by Audeering [24], the Technical University of Munich, Germany). Each feature is representative of the whole audio segment, with several different descriptors being used to unify the temporal information (e.g., mean over windows, standard deviation, etc.). The INTERSPEECH 2016 configuration [25] was employed, with a grand total of 6373 features being extracted, covering the vast majority of relevant domains in voice analysis, including time, spectrum, cepstrum, RASTA [26], prosody and perceptual features. 

Extracted features were reduced with the aid of a Correlation-based Feature Selector (CFS) [27]. It is a supervised method based on the identification of the best subset following the maximum-relevance, minimum-redundancy principle, using correlation as a metric according to the following formula:

(2) $$M_{S}=\frac{k*\overset{-}{r_{fc}}}{\sqrt{k+k\left(k-1\right)*\overset{-}{r_{ff}}}}$$

Where *k* is the number of features in a subset *S*, $\overset{-}{r_{fc}}$ is the average correlation between features and the class label, and $\overset{-}{r_{ff}}$ is the average correlation between pairs of features in the subset. A Forward Greedy Stepwise search method is used to identify the optimal subset, which contains a non-predetermined number of features.

#### 2.3.2. Naïve Bayes Classifier

A multi-class Naïve Bayes (NB) was trained on the selected features; it is a supervised probabilistic classifier based on Bayes’ Theorem to compute the posterior probability of a set of features pointing to a certain class, which is directly proportional to the prior class probability and the measured likelihood of each feature [28]. Working with numeric, non-discretized features, a Gaussian curve fit was employed to compute the posterior probability for each feature. A 10-fold cross validation was used to assess accuracy values: the whole dataset was divided into ten complementary folds, sampled without repetition, so that ten 9-vs-1 comparisons could be performed. Each speaker/class was split individually, so that the 90% of its samples made the training set and different 10% folds made the cross-validation sets. The final results were the average of these ten comparisons, so that the whole dataset was eventually used as validation. Classification and feature selection were implemented using Weka^®^ (University of Waikato, New Zealand [29]).

## 3. Results

Results of the DL and ML-based approaches are here reported. Since many different architectures and experiments were carried out with CNNs, only several relevant ones, which bring to the highest accuracies we found, are reported in Table 2, comparing our custom architecture CNN1 to AlexNet. More information about additional solutions that have been attempted with no relevant results are given in the Discussion. As an example, we employed a GoogleNet trained on the same variations of input data as reported before (grayscale/colored spectrograms/MFCC), but we did not reach satisfactory results, with the average accuracy being 73.8% for MFCC. 

**Figure 6:**
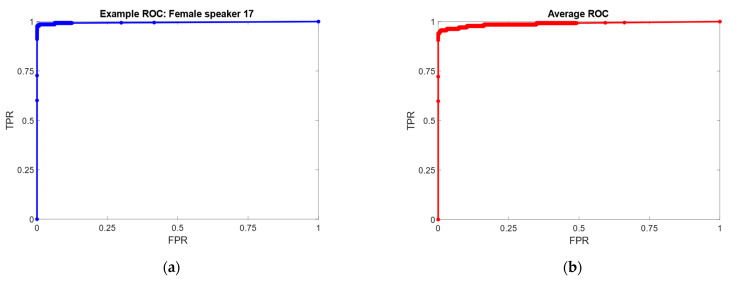
(**a**) Example ROC curve, by female speaker. AUC = 0.991; (**b**) Average ROC curve computed by macroscopically averaging FPR and TPR values for each one-vs-all comparison. Average AUC is 0.985. FPR (x-axis) = False Positive Rate (or 1–Specificity), TPR (y-axis) = True Positive Rate (Sensitivity).

Table 3 presents the results obtained with the Naïve Bayes, also reporting data on False Positives (FP) over all 58 speakers considered as well as the AUC (Area Under the ROC Curve) computed for each speaker, in a one-vs-all fashion, averaged on each fold of the cross-validation. ROC curves can be visualized in Figure 6, with an example one by a female speaker along with the macro-average curve, computed finding values for FPR (x-axis) and TPR (y-axis) by averaging the values of all one-versus-all binary classification problems [30]. 

## 4. Discussion

The results initially show the feasibility of SR with both DL and ML-based approaches on the DEMoS dataset, with the best-performing method being the CNN1 architecture trained on grayscale spectrograms, reaching a 90.15% accuracy. 

Many CNN configurations were attempted, with the CNN1 architecture being the best-performing custom one and AlexNet providing the best results for pre-trained nets. Both AlexNet and our custom CNN1 overcame GoogleNet in terms of average accuracy for all tasks. Results between colored and grayscale images are comparable, with grayscale interestingly bringing the best overall results for spectrograms. Although it is evident that there was a small loss of information in terms of amplitude resolution in the passage between 3-channel (RGB) colored images and grayscale, grayscale sometimes appeared to bring better performances. This is not uncommon in CNN, and the usual accepted answer is to be found in the smaller number of variables that the naïve nets have to learn with grayscale inputs. In fact, at the negligible price of a certain loss of resolution, the amount of learnables is reduced by a factor of three, which makes the architecture much faster and less prone to underfitting. Audio signals can indeed be considered as “more complex/variable” than images, at least on the point of view of temporal evolution; however, spectrograms embed frequency and time-related information in a compact and complete graphical way which has been used with CNNs for many years, often providing state-of-the-art results. For audio tasks, relevant information can be found in trends within pitch and frequency content, which appear as pseudo-straight lines and darker/lighter areas on the spectrograms. With these premises, it also becomes explainable the reason why MFCC-graphs appear to perform better with colored images: with only 32 coefficients, the amount of information that could “confuse” the nets is less and the added resolution can be handled. 

Additional experiments have been carried out by pre-training custom architectures and other transfer-learnt nets with spectrograms/MFCC and then training on the other image inputs (e.g., net pre-trained on the spectrograms, re-trained on MFCC’s). This did not bring relevant improvements to the accuracies, although in general they were comparable. 

Although spectrograms bring better accuracies with respect to MFCC, possibly due to their un-discretized nature containing more information, tests on the training time show that, due to their size, MFCC’s take roughly half the amount of time for training. Specifically, for the CNN1 architecture, an average training time of 29 min is needed for MFCC versus 72.5 min for spectrograms, on the same Windows computer equipped with a 6th generation Intel i7^®^ (Santa Clara, CA, USA)processor with 16GB RAM. 

As far as Machine Learning is concerned, the “common” pipeline of extraction-selection-classification is employed with a Naïve Bayes classifier. The accuracy is slightly lower than CNN, but ML holds several advantages: after a proper feature selection, the training time is much shorter. Moreover, the results are much more interpretable, since they are computed on real acoustic features. 

The generalization power of the NB is confirmed by very high AUC values for each speaker, and the False Positive rate which is 0.2% on average. 

**Figure 7:**
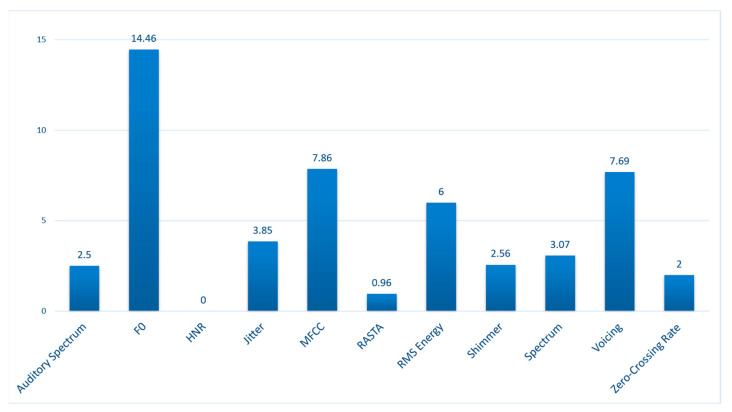
Distribution of each feature domain within the selected features. It is quantified as a percentage of the original number of features per domain in the INTERSPEECH 2016 feature set. Abbreviations: HNR = Harmonic-To-Noise ratio, MFCC = Mel-Frequency Cepstral Coefficients, RASTA = Relative Auditory Spectrum, RMS = Root-Mean Square.

As far as acoustic features are concerned, we chose to employ a comprehensive set that included the vast majority of features considered useful in speech analysis, hence our choice to use the INTERSPEECH 2016 feature set. In accordance with the principle of the Curse of Dimensionality, the original number of features, intentionally large as to cover all possibilities, was then algorithmically reduced using a CFS [31]. Although MFCC are the most widely employed domain for SR, especially with the introduction of the concept of i-vectors, it is undoubtable that many other features can indeed define the peculiarities of a speaker’s voice. F0 and its variations are deeply related to intonation, whereas the amount of short breathing pauses, evaluated by means of the Voicing Probability, are shown to be able to classify between speakers [32]. The importance of said features is confirmed by the results of the feature selection, that shows the number of selected features being 3.45% of the original number. The distribution of the main domains of the selected features, computed as a percentage of their original number is reported in Figure 7. Each domain embeds many different linear (e.g., maximum), differential (e.g., delta coefficients), statistical (e.g., standard deviation) descriptors.

F0-related measures appear as the most relevant, which is in line with the fact that some of the most immediate characterizing features for SR tasks, even when performed by ear, can be found in someone’s pitch and intonation (pitch variability) [7]. MFCC, which are frequently the standard in SR, provide around half the score than F0 but are indeed assessed as relevant, which is in line with the literature and with common methodologies. As a close third come the voicing-related features, whose score is almost the same as MFCC, although them being based on Yeldener’s principle for assessing the amount of “voiced” and “unvoiced” sections in a signal [33]. Since each audio is a small segment that does not contain long silences, differences in voicing could at most account for the number of small pauses and breath. Since overfitting happens when a classifier model adapts too closely to the training data, the main metrics that allow to minimize it and to consider its effects negligible are to be found in the usage of datasets with large cardinality and heterogeneous characteristics, a thorough validation procedure and the usage of the right features. We addressed all this by using a text-independent, manually segmented, externally validated dataset with a large number of samples (8869), and by employing cross validation for testing, which allowed to produce generalized accuracy metrics that eventually spanned through the whole dataset. The presence of typical features recognized as a standard for speaker recognition among the selected ones could also be seen as a further confirmation that the models are indeed searching for the right characteristics for classification. Moreover, high AUC values account for the generalization power of the Naïve Bayes classifier. 

Interestingly, higher FP rates are found for female speakers, which is in line with the gender unbalance within the DEMoS dataset, which has fewer female examples for the model to learn from. 

This, along with the small number of speakers, can be considered as one of the limitations of the present study. However, the amount of audio files, which is almost 9000, is high enough to grant a certain degree of generalization for the validation procedures. 

For the present study, no data augmentation was applied to the audio samples. However, it has been shown that even simple augmentation techniques such as noise addition or time/frequency masking could help improve classification accuracy and generalization power. Additionally, although we did experiment on more than one pre-trained nets for transfer learning (namely, AlexNet and GoogleNet), there are many other viable ones that brought interesting results in the field of voice analysis, such as Inception V2 [34] which is an evolution of GoogleNet, Xception [35] and ResNet50 [36] which we used successfully in other voice analysis tasks [2]. Although CNN (or Deep Neural Networks) have proven their worth in audio analysis, Recurrent Neural Networks (RNN) and Long-Short-Term Memory (LSTM) networks, especially fit for time series analysis, still provide relevant results [37]. However, on the one hand the state-of-the-art in speaker recognition, as detailed by Trabelsi et al. [38], is either derived from “traditional” methods or Deep Neural Network-based techniques such as DeepSpeech, and these are the two methodologies that we also employed in the present study. On the other hand, having already performed a thorough segmentation that led to a dataset made of short and compact speech chunks, time series analysis is inherently less viable. Additionally, we did not experiment on more complex “ensembled” methodologies that add further algorithms to DL classifiers; nor did we explore other ways such as NMF or GMM [39]. 

The (elicited) emotional content of the DEMoS dataset could be considered as a further complication, although its effects are mitigated by the audio segmentation procedure and do also reflect reality; a SR task must take into account the eventual, unpredictable emotional state of the speaker at any given moment. 

## 5. Conclusions

In this work, high-level AI methodologies for Speaker Recognition are explored without building speaker-specific models. Several CNN architectures are experimented, with a shallow, custom one providing the best results (90.15% accuracy) along with AlexNet pre-trained on the ImageNet dataset. Grayscale spectrograms provide the highest accuracies, even topping MFCC-graphs. A Naïve Bayes trained on selected acoustic features is compared, with its accuracy coming short at 87.09% while being a much lighter model. Acoustic features within the domain of pitch/F0, MFCC and voicing probability are assessed as the most effective for classification.

## Figures and Tables

**Table 1:** Distribution of the instances (i.e., audio segments) with respect to the speaker sex and emotion for the subset of the DEMoS dataset used in the present study.

Emotion	Speaker Sex		Tot.
	Male	Female	
Anger	868	449	1317
Guilt	638	338	976
Fear	675	360	1035
Surprise	611	313	924
Disgust	1003	542	1545
Happiness	918	483	1401
Sadness	890	453	1343
Neutral	220	108	328
Tot.	5823	3046	8869

**Table 2:** Accuracies for MFCCs and Spectrograms using our custom CNN1 and AlexNet.

Accuracy	Input	Net
90.15%	Spectrogram, grayscale	CNN1
88.85%	Spectrogram, colored	CNN1
82.27%	MFCC, grayscale	CNN1
83.17%	MFCC, colored	CNN1
83.43%	MFCC, colored	AlexNet
89.28%	Spectrogram, grayscale	AlexNet

**Table 3:** Results of the Naïve Bayes-based ML approach. FP = False Positive, TP = True Positive, avg. = Average, AUC = Area Under the ROC Curve, RMS = Root Mean Square.

Metric	Value
FP range (min-max)	0.000–0.010
FP (weighted avg.)	0.002
TP range (min-max)	0.364–0.978
TP (weighted avg.)	0.871
AUC (weighted avg.)	0.985
Precision (weighted avg.)	0.875
Recall (weighted avg.)	0.871
F1 Score (weighted avg.)	0.872
Mean Absolute Error	0.0044
RMS Error	0.0655
Relative Absolute Error	13.09%
Accuracy	87.09 %

## Data Availability

The DEMoS dataset employed in this study is available upon request at the link https://zenodo.org/record/2544829.

## Supplementary Materials

The following supporting information can be downloaded at: https://www.mdpi.com/article/10.3390/s23073461/s1, Table S1: confusion matrix, Table S2: full list of selected features.
